# Paradoxical Reactions to Midazolam in a Term Parturient After Intravenous Sedation During Cesarean Section

**DOI:** 10.7759/cureus.17678

**Published:** 2021-09-03

**Authors:** Sengottaian Sivakumar, Roni Mendonca, Don Demeterio, Micheal Girshin

**Affiliations:** 1 Anesthesiology, Metropolitan Hospitals, New York, USA; 2 Anesthesiology and Perioperative Medicine, Metropolitan Hospitals, New York, USA

**Keywords:** midazolam, paradoxical reaction, cesarean section, flumazenil, epidural anesthesia, cystic fibrosis carrier

## Abstract

Propofol and midazolam are commonly used drugs in procedural sedation. Midazolam is widely used for its five principal pharmacologic effects: anxiolysis, sedation and hypnosis, anticonvulsant actions, spinal cord-mediated skeletal muscle relaxation, and anterograde amnesia. Increased talkativeness, emotional release, excitement, and excessive movement are the common paradoxical reactions to all kinds of benzodiazepines, which are reported since the introduction of chlordiazepoxide (Librium), the first benzodiazepine in 1955. In the United States, sedation with a combination of midazolam with opioids accounts for approximately 75% of routine procedural sedations. Most cases are distinctive. However, some data indicate that these reactions are due to serotonin imbalance, a central cholinergic effect, or a reflection of genetically determined variability in benzodiazepine receptor density or affinity (isoreceptors) throughout the brain. The idea of isoreceptors is comparable to that of isoenzymes like genetic variants of pseudocholinesterase. We report a case in which midazolam administration resulted in paradoxical reactions, which manifested as profound delirium with extrapyramidal symptoms after cessation of propofol sedation in a term parturient during cesarean section. This case report describes paradoxical reactions to benzodiazepines in a term parturient promptly reversed with a small dose of flumazenil. Even though paradoxical reactions to benzodiazepines have low prevalence and are not life-threatening, they have to be treated promptly with flumazenil. Therefore, anesthesiologists performing procedural sedation should be aware of untoward reactions and be prepared to manage them promptly.

## Introduction

Propofol and midazolam are commonly used drugs in procedural sedation. Midazolam is widely used for its five principal pharmacologic effects: anxiolysis, sedation and hypnosis, anticonvulsant actions, spinal cord-mediated skeletal muscle relaxation, and anterograde amnesia. Increased talkativeness, emotional release, excitement, and excessive movement are common paradoxical reactions to all kinds of benzodiazepines, which have been reported since the introduction of chlordiazepoxide (Librium), the first benzodiazepine in 1955. In the United States, sedation with a combination of midazolam with opioids accounts for approximately 75% of routine procedural sedation for esophagogastroduodenoscopies and colonoscopies [1]. Most cases are distinctive. However, some data indicate that these reactions are due to a genetic link [2]. We report a case in which midazolam administration resulted in paradoxical reactions, which manifested as profound delirium with extrapyramidal symptoms after cessation of propofol sedation in a term parturient.

## Case presentation

 A 37-year-old Gravida 3 Para 0 Hispanic female parturient at 40 weeks of gestation presented with lower abdominal pain and regular contractions. Her past medical history was significant for cystic fibrosis carrier state and gestational diabetes mellitus. The patient had no history of psychiatric illness, substance abuse, or seizure disorder. Throughout her antenatal period, she did not take any medications except for prenatal vitamins. As her labor pains progressed, she requested labor epidural analgesia. Preprocedural examination revealed body mass index (BMI) 29.49 kg/m^2^, pulse rate 90/minute, blood pressure 96/66 mmHg, oxygen saturation 99%, and respiratory rate 14/minute. With the patient in a sitting position, the L3-4 interspace was identified, and the procedure was carried out with standard sterile precautions. With an 18G Tuohy needle, the epidural space was reached at 4 centimeters from the skin by the loss of resistance to saline technique. A 20 G multi-orifice epidural catheter threaded up to a length of 10 centimeters at the skin level. After a negative test dose, ropivacaine 2 mg/ml epidural infusion started at the rate of 10 ml/hour. Her pain relief was excellent, and she continued to be in labor for 29 hours post epidural placement.

Due to arrest of descent, an emergent cesarean section was called, the patient was moved to the operating room, and standard American Society of Anesthesiologists (ASA) monitors were connected. Epidural fentanyl 100 mcg bolus along with three separate doses of 5 ml 2% lidocaine with epinephrine 1: 100,000 was given at three-minute intervals. The sensory level of the blockade was T4 before incision. A single fetus was delivered 15 minutes after the skin incision. APGAR scores at one, five, and 10 minutes were within normal limits. Umbilical cord arterial blood gas showed normal PH.

The surgeon notified accidental posterior cystotomy measuring approximately 1 centimeter, and the urology team performed cystotomy repair. Due to the patient's increasing anxiety, midazolam 2 mg intravenous bolus was administered and 40 mcg/kg/min propofol infusion was started and continued till the end of the surgical procedure. The dose of propofol sedation was titrated to effect and was maintained between 40 and 60 mcg/kg/min. She also received 100 mcg intravenous fentanyl and another 2 mg midazolam bolus one hour after the first dose. In addition, the operating surgeon requested oxytocin 30 IU in 500 ml of lactated ringer premix solution, intramuscular methergine, and 250 mcg of intrauterine carboprost for uterine atony. Other medications used during the intraoperative period were 4 mg of ondansetron and 4 mg of dexamethasone. At the end of the surgery, propofol infusion was stopped. When the patient emerged from sedation, she developed intermittent jerky movements of her upper extremity, which lasted for five seconds, followed by aggressive behavior, irrational talking, and restlessness. Her state of aggression and excitation lasted for over 15 minutes. Flumazenil 0.2 mg intravenous reversed these behaviors. Postoperative head CT and EEG were done to rule out underlying focal seizures or any intracranial pathology. Even though her aggression came down drastically after flumazenil, her elated mood lasted for almost four to five hours after she emerged from sedation. She could not recollect that she gave birth to a baby. Breastfeeding was withheld for four hours after birth due to the mother's mental instability. The patient was discharged from the hospital on the fifth postoperative day.

## Discussion

Delirium is an unfathomable clinical syndrome characterized by an acute and reversible failure of the brain's essential cognitive and attentional functions. Delirium can be either agitated (hyperactive type), lethargic (hypoactive type), or alternate between these two subtypes (mixed type). Among pregnant women, the postpartum period is the most prevalent stage for the occurrence of mental disturbances. It is contributed by multiple factors and is generally associated with physiologic and pharmacologic disruptions occurring in the immediate perioperative period. Differential diagnosis of postoperative delirium is extensive. Major causes include drug reactions, intractable pain, acute pathological states such as cerebrovascular events causing changes in brain perfusion or oxygenation, exacerbation of preexisting neurologic or psychiatric illness, and psychological stress related to surgery itself.

Among the drugs given to this patient, intravenous propofol, intravenous and epidural fentanyl [3], midazolam [4-7], and ondansetron [8] are all capable of producing delirium and excitatory symptoms. Paradoxical reactions are common in patients with alcohol abuse disorder or mental health condition that causes extreme mood swings [7]. Our patient had no prior psychiatric or neurological illness. Her intraoperative glucose levels were also within normal limits. No hypoxic or hypotensive episodes were noted during the perioperative period, resulting in cerebral hypoxia. As the patient had a working epidural block supplemented at regular intervals, intractable pain may not be the reason for her delirium. These paradoxical reactions can also be attributed to propofol [9], but it was readily reversed with flumazenil, which quickly points towards the diagnosis of midazolam-induced paradoxical reactions.

As midazolam readily crosses the placenta, it is associated with severe respiratory depression in newborns. During cesarean section, it is commonly given for anxiolysis after delivery of the fetus. Unfortunately, some patients experience paradoxical reactions to benzodiazepines. These may include agitation, hallucinations, restlessness, disorientation, uncontrollable crying or verbalization, involuntary movements, self-injury, and aggressive or violent behavior, which sometimes requires restraints. Usually, they manifest in five minutes after administration, and these reactions are termed paradoxical or disinhibitory reactions. These reactions are more common in the pediatric population [10].

Nearly all these reactions are preceded by a short period of apparent sedation, after which the patient is seen in a state of intense agitation. Reactions do not affect the vital signs and are usually not recalled by the patient. However, it creates an unpleasant memory for the caregivers surrounding the patient and can also cause bodily harm from physical injuries due to agitation and aggressive behavior. Although the exact mechanism of paradoxical reactions is unknown, it may be related to the central cholinergic effect or serotonin imbalance or a reflection of genetically determined variability in benzodiazepine receptor density or affinity (isoreceptors) throughout the brain [2,7]. The idea of isoreceptors is comparable to that of isoenzymes like genetic variants of pseudocholinesterase.

Midazolam is metabolized by hepatic and small intestine cytochrome CYP3A4 enzymes to active and inactive metabolites. Cystic fibrosis carriers have one copy of the mutated cystic fibrosis gene and increased incidence risk of a wide range of cystic fibrosis-related conditions. Cystic fibrosis patients have increased intestinal CYP3A4 activity, affecting the metabolism of orally administered midazolam [11]. The activity of CYP3A4 enzymes was not studied in cystic fibrosis carriers.

The management of these reactions should be done systematically. Soothing and comforting of the patient is instituted. If the patient is unconstrained, combative, and confused, a diagnosis of paradoxical reaction should be weighed consistently. An initial dose of flumazenil (0.1-0.2 mg) should then be administered intravenously [7]. Furthermore, the same dose can be repeated after 60 seconds to a maximum dose of 1 mg. The onset of flumazenil's effect is two to three minutes, and its peak effect occurs at five to six minutes. Flumazenil should be used judiciously if the patient has a history of seizures controlled with benzodiazepines. Postoperative monitoring of the patient is crucial because paradoxical reaction might reappear as flumazenil has a shorter half-life than midazolam [7]. In such cases, a continuous infusion may be required at the dose is 0.1- 0.4 mg per hour. For pediatric cases, reversal with flumazenil has been attained with doses of 0.01 mg/kg [12]. Flumazenil can cause seizures and cardiac arrhythmias, and it has to be used with caution in preexisting seizure disorder and cardiac arrhythmia patients [13]. Even though there are concerns for breastfeeding for few hours after the last dose of flumazenil [14], in this case, breastfeeding was withheld for five hours after birth due to the mother's mental instability.

## Conclusions

In summary, this case report describes paradoxical reactions to benzodiazepines in a term parturient promptly reversed with a small dose of flumazenil. Even though paradoxical reactions to benzodiazepines have low prevalence and are not life-threatening, treatment with flumazenil should be strongly considered. Therefore, anesthesiologists performing procedural sedation should be aware of the untoward reactions and should be prepared to manage them promptly.
